# The crisscross between p53 and metabolism in cancer

**DOI:** 10.3724/abbs.2023109

**Published:** 2023-06-19

**Authors:** Youxiang Mao, Peng Jiang

**Affiliations:** 1 State Key Laboratory of Molecular Oncology School of Life Sciences Tsinghua University Beijing 100084 China; 2 Tsinghua-Peking Center for Life Sciences Beijing 100084 China

**Keywords:** p53, metabolic reprogramming, cancer, metabolite sensing, gene regulation

## Abstract

As the guardian of the genome, p53 is well known for its tumor suppressor function in humans, controlling cell proliferation, senescence, DNA repair and cell death in cancer through transcriptional and non-transcriptional activities. *p53* is the most frequently mutated gene in human cancer, but how its mutation or depletion leads to tumorigenesis still remains poorly understood. Recently, there has been increasing evidence that p53 plays a vital role in regulating cellular metabolism as well as in metabolic adaptation to nutrient starvation. In contrast, mutant p53 proteins, especially those harboring missense mutations, have completely different functions compared to wild-type p53. In this review, we briefly summarize what is known about p53 mediating anabolic and catabolic metabolism in cancer, and in particular discuss recent findings describing how metabolites regulate p53 functions. To illustrate the variability and complexity of p53 function in metabolism, we will also review the differential regulation of metabolism by wild-type and mutant p53.

## Introduction

The Warburg effect, a common characteristic of cancer cells, is defined as a high rate of aerobic glycolysis [1]. It has become increasingly clear that cancers have diverse metabolic profiles, with many relying primarily on macromolecule biosynthesis and ATP production. This is because rapidly proliferating cancer cells require large amounts of glucose to fuel anabolism and are more likely to exhibit Warburg effect phenomena [ 2, 3] . In addition to glycolysis, numerous studies suggest that glutaminolysis, lipid, amino acid and ferroptosis are reprogrammed in cancer cells in response to growth stimulation or under certain stress conditions [ 4‒ 6] .

The tumor suppressor p53, discovered in 1979 [ 7‒ 10] , is the most widely known gene associated with cancer [11]. There are currently at least 8 p53 activator compounds in clinical trials, and targeting the interaction between p53 and MDM2 and reactivating the wild-type functions of mutant p53 are two major strategies in drug discovery [12]. p53 responds to a considerable number of extrinsic and intrinsic insults, including DNA damage, oncogene activation, hypoxia, heat and cold shock, protein unfolding/misfolding stress, nutrient deprivation, whole genome duplication and telomere shortening [ 13‒ 16] . Through transcriptional and non-transcriptional activities to regulate specific gene expression in response to various stimuli, p53 plays a critical role in the control of cell proliferation, senescence, DNA repair and cell death. However, over the last decade a large body of evidence has emerged that challenges the simplistic interpretation of p53 as a tumor suppressor. Accumulating evidence suggests that p53 also regulates cellular metabolism, including glycolysis, the TCA cycle, oxidative phosphorylation, glutaminolysis, the pentose phosphate pathway, lipid metabolism, nucleotide synthesis, iron metabolism, and polyamine biosynthesis [ 17, 18] . And, metabolic regulation appears to be central to the tumor suppressor function of p53. Moreover, unlike wild-type p53, mutant p53s have completely different regulatory functions in cancer metabolism, and understanding these may provide significant insights into how p53 suppresses tumors.

## The Emerging Role of p53 in Anabolism and Catabolism

**Figure 1:**
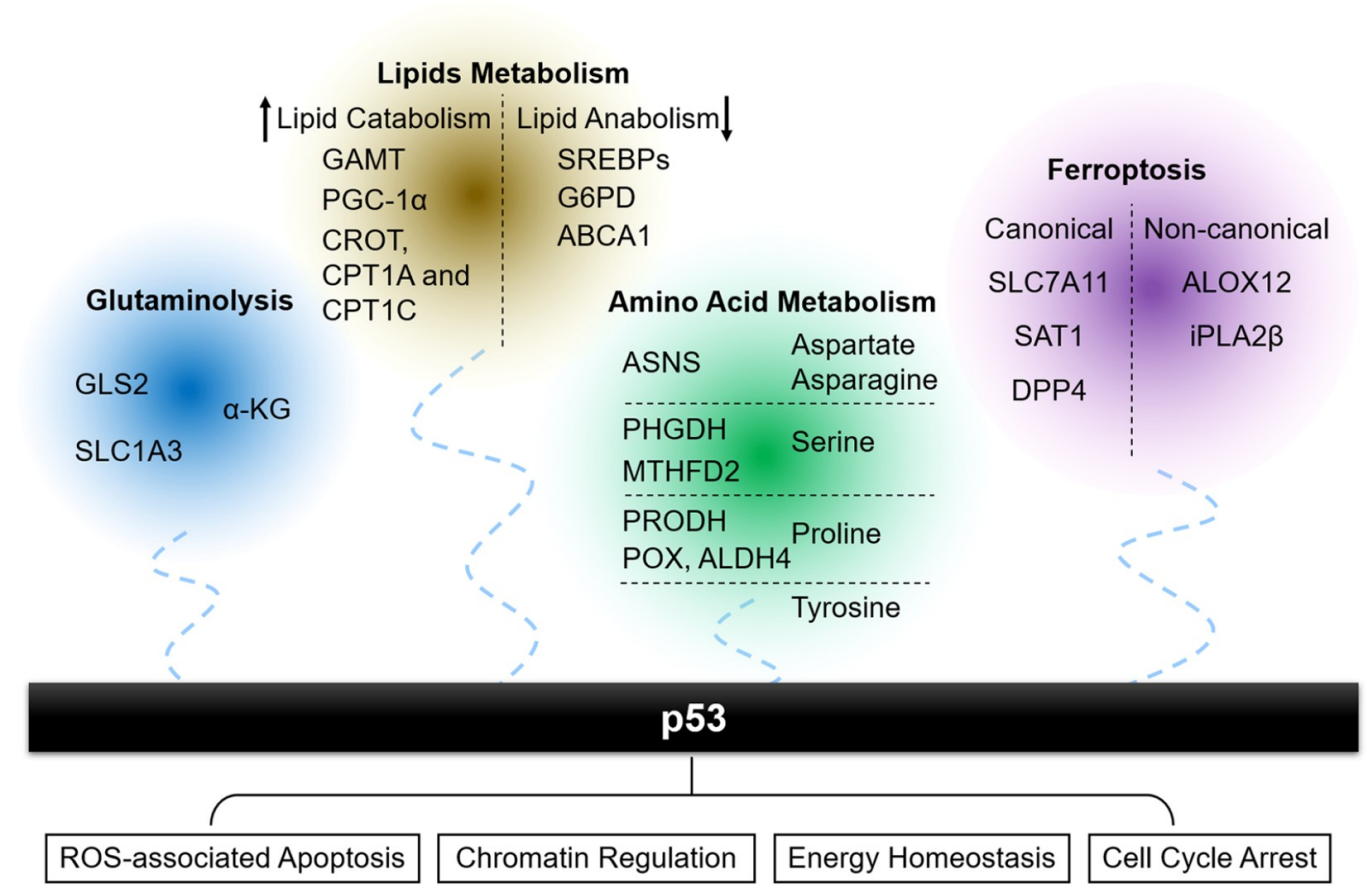
Figure 1 The regulatory role of p53 in anabolism and catabolism p53 plays a pivotal role in regulating glutaminolysis, lipids metabolism, amino acid metabolism and ferroptosis to dominate ROS-associated apoptosis, chromatin regulation, energy homeostasis and cell cycle arrest.

p53 directly or indirectly regulates metabolic homeostasis and normally directs anabolism towards catabolism ( Figure 1), by regulating key metabolic pathways, including central carbon metabolism, lipid and amino acid metabolism, ion metabolism, ammonia detoxification and polyamine biosynthesis. When certain nutrients are scarce, p53 maintains the energy required for cell survival and basic life activities, as well as the prerequisite substances for the synthesis of biological macromolecules. As the regulation of p53 and glycolysis has been systematically reviewed [19], we will not discuss it here.

### p53 and glutaminolysis

Glutamine, the most abundant circulating amino acid in serum, is a major carbohydrate source for the TCA cycle and the malate-aspartate shuttle [20]. Glutamine is essential for maintaining mitochondrial metabolism, ROS scavenging, nucleotide synthesis, protein synthesis and activation of cell signaling. The process of converting glutamine to glutamate through a series of biochemical reactions is called glutaminolysis [21]. A key enzyme in glutamine metabolism is mitochondrial glutaminase (GLS), which converts glutamine to glutamate and regulates the synthesis of glutathione (GSH) to support antioxidant defenses and energy production. Glutamate is then converted to 2-oxoglutarate (α-KG, also known as α-ketoglutarate) by glutamate dehydrogenase (GLUD) or a group of transaminases including glutamate oxaloacetate transaminase (GOT), glutamate pyruvate transaminase (GPT) and phosphoserine transaminase (PSAT) [22]. Interestingly, some cancer cells have unusually high activity of glutamine synthetase (GS), which converts glutamate to glutamine [23].

Glutamine metabolism is altered in cancer development. Both the uptake of glutamine and the rate of catabolism of glutamine to produce ATP and lactate are increased in cancer cells [24]. There are two different phosphate-activated GLS isoforms in mammals, GLS1 (kidney type) and GLS2 (liver type). p53 controls glutamine catabolism by regulating the expression of GLS2. GLS2 reduces cellular sensitivity to ROS-associated apoptosis in a p53-dependent manner [25]. Moreover, p53 plays a regulatory role in adjusting the TCA cycle activity by repressing the expression of malic enzymes, leading to accelerated cell senescence [26]. By comparing 409 breast cancer tissue samples, researchers discovered that the expression of SIRT4 is significantly higher in the p53-positive cancer tissues [27]. Loss of the mitochondrial sirtuin-SIRT4 leads to increased glutamine catabolism through ADP-ribosylation and hence repression of GLUD [ 28, 29] . Interestingly, in pancreatic ductal adenocarcinoma (PDAC), inhibition of glutamine metabolism by SIRT4 promotes phosphorylation of p53, leading to reduced tumor development [30].

In addition, p53 rewires glucose and glutamine metabolism to favor the accumulation of α-KG at the expense of succinate. Restoring p53 function in cancer cells derived from KRAS-mutant mouse models of PDAC leads to the accumulation of the metabolite α-KG as a cosubstrate of the methylcytosine dioxygenases TETs, resulting in increased 5-hydroxymethylcytosine (5-hmC) of chromatin [31]. p53 induces transcriptional programs characteristic of premalignant differentiation, and this effect can be partially recapitulated by the addition of α-KG. Loss of p53 prevents these metabolic effects and allows the transition to more aggressive and less differentiated carcinomas, characterized by reduced α-KG-dependent activity [32]. A double knockout mouse model showed that genomic integrity requires the cooperation of p53 and SIRT1 [33]. Mechanically, SIRT1-mediated p53 deacetylation regulates cell fate under oxidative stress [34]. Thus, p53 links cancer-cell metabolism between chromatin regulation and tumor-cell fate.

Cancer cells are often unable to access sufficient nutrients and oxygen due to inadequate blood supply, such as the depletion of glutamine in developing tumors. p53 can recruit certain proteins to boost its metabolic function, and cell lines differ in their sensitivity to glutamine starvation depending on the activity of p53 [35]. It is interesting to understand how cancer cells withstand glutamine deprivation in order to survive and proliferate. A recent study has shown that p53 can help cancer cells adapt to glutamine deprivation, as p53-null cells fail to maintain the TCA cycle in response to glutamine deprivation. Mechanically, p53 maintains the utilization of aspartate to support cells during glutamine deprivation by promoting the expression of SLC1A3, an aspartate/glutamate transporter [36], which is pivotal for maintaining electron transport chain and TCA activity, promoting *de novo* glutamate, glutamine, and nucleotide synthesis to rescue cell viability. Furthermore, during glutamine deprivation, TRIAP1 (TP53-regulated inhibitor of apoptosis 1) depletion confers robust p53-mediated resistance to metabolic stress [37]. Viewed in this light, p53 can play both good cop and bad cop in glutaminolysis.

### p53 controls lipid metabolism

Lipids are composed of many types of hydrophobic molecules, including fatty acids, phospholipids, sterols and glycolipids [38]. Lipids play a role as membranes components, energy source, and signaling messengers [ 39, 40] . Cancer cells tend to boost *de novo* lipid synthesis and increase lipid uptake to meet rapidly proliferating demands [ 6, 41] . However, these effects can be kept under control by p53. p53 transactivates key proteins in lipid catabolism, thereby depriving cancer cells of lipid. A study examining the expression pattern of fatty acid metabolism genes in breast cancer patients showed that patients with wild-type p53 had significantly increased levels of FABP4, PLIN1 and MGLL and decreased level of FABP5 compared with mutant p53 [42]. p53 regulates fatty acid metabolism by activating and regulating the expression of guanidinoacetate methyltransferase (GAMT), which promotes fatty acid oxidation (FAO) and creatine biosynthesis and plays an essential role in maintaining energy homeostasis during glucose deprivation [43]. Mechanically, under starvation conditions, p53-dependent activation of GAMT can induce energy expenditure and promote apoptosis. PGC-1α, a regulator of lipid metabolism, binds to the promoter of p53 to promote cell cycle arrest and ROS scavenging in response to glucose starvation [44]. In addition, p53 also transcriptionally upregulates the expressions of three carnitine acyltransferases-carnitine octanoyltransferase (CROT), CPT1A and CPT1C which facilitate the transport of fatty acids into mitochondria [45], malonyl-CoA decarboxylase (MCD) [46] and lipin 1 (LPIN1) in response to nutrient deprivation. In colorectal cancer, p53 also promotes peroxisomal fatty acid β-oxidation, which increases cytosolic acetyl-CoA levels and acetylation of the enzyme 5-aminoimidazole-4-carboxamide ribonucleotide formyltransferase/IMP cyclohydrolase (ATIC), the enzyme that catalyzes the last two steps of purine synthesis [47].

Accumulating evidence shows that p53 also inhibits lipid anabolism, mainly through indirect regulation. p53 transcriptionally inhibits the expression of sterol regulatory element-binding proteins (SREBPs), key transcription factors that target lipogenesis genes [48]. In addition, p53 suppresses *de novo* fatty acid synthesis by reducing NADPH levels by inhibiting the rate-limiting enzyme of the PPP pathway–Glucose-6-phosphorylate dehydrogenase (G6PD) [49]. p53 also mediates cholesterol metabolism through the mevalonate pathway, which is involved in the biosynthesis of cholesterol and non-sterol isoprenoids. For example, in a mouse model of liver cancer, p53 upregulates ABCA1, which mediates retrograde sterol movement from the plasma membrane to the ER and induces repression of SREBP-2 maturation [ 50, 51] .

Due to the importance and frequent alteration of lipid metabolism in cancer cells, the fatty acid synthesis pathway may be an important pathway to exploit clinically in the diagnosis, treatment and prevention of cancer, and the p53-lipid axis may play a role in tumor suppression or in maintaining the balance between the glycolytic and respiratory pathways and counteracting the metabolic shift in tumorigenesis.

### p53 in amino acid metabolism

There are 20 canonical amino acids in humans, which combine into peptide chains to form the building blocks of proteins. Here we will only discuss the amino acids regulated by p53, in particular aspartate, asparagine, serine, tyrosine and proline.

Asparagine can be produced either by protein breakdown or directly from aspartate. Asparagine synthetase (ASNS) is responsible for the conversion of aspartate and glutamine to asparagine and glutamate. Notably, recent studies showed that ASNS inhibition renders tumor cells more susceptible to glutamine depletion-induced apoptosis and that asparagine supplementation sufficiently reverses this effect independently of TCA cycle anaplerosis [52]. Notably, there is convincing evidence that the level of asparagine is significantly higher in the serum of p53 ^-/-^ mice than in p53 ^+/+^ ones and that the high level of asparagine increases lymphoma cell proliferation in p53 ^-/-^ mice. Mechanistically, p53 transcriptionally controls ASNS expression and loss of p53 disrupts aspartate-asparagine homeostasis and leads to unexpected asparagine synthesis and accumulation in the microenvironment [53]. This study provided a clue to understanding why p53-deficient mice predominantly develop lymphomas [54].

Rapidly proliferating cells have a high demand for serine, which can be synthesized *de novo* or taken up from the environment, and is generally used for protein synthesis and the production of anabolic intermediates, including sphingolipids, nucleotides, NADPH and glutathione (GSH) [55]. Importantly, Dr. Vousden and her colleagues [56] described a role for p53 in supporting cell survival during serine starvation, linking p53 to serine metabolism and cell fate determination through p21-mediated transient cell cycle arrest. These findings also suggest a potential clinical treatment for p53-deficient tumors by removing serine. PHGDH, a key enzyme of serine synthesis, is transcriptionally repressed by p53 in melanoma cells cultured in complete medium [57]. In this context, cancer cells may benefit from retaining p53 because it enables them to adapt to serine-deficient conditions and promote their survival [19]. Serine can be metabolized to glycine, both of which are essential carbon sources for purines. Serine supports purine synthesis through a single-carbon metabolic pathway as a carbon donor, of which methylenetetrahydrofolate dehydrogenase (MTHFD2) is a major enzyme that is frequently overexpressed in human cancers. One-carbon metabolism, which involves both the folate and methionine cycles and allows cells to generate and utilize one-carbon units for the biosynthesis of important anabolic precursors and for methylation reactions, has been of intense interest in recent years. MTHFD2 is an NAD(P) ^+^-dependent, bifunctional mitochondrial folate enzyme. It catalyzes the interconversion of 5,10-methylene-THF to 10-formyl-THF and produces formate for purine synthesis [58]. In many types of cancer, MTHFD2 expression is highly positively correlated with p53 deletion or mutation and has recently been found to be a direct transcriptional target of p53. Interestingly, inhibition of MTHFD2 not only restricts cellular NADPH synthesis and DNA synthesis, but also selectively causes DNA damage and apoptosis in p53-deficient or mutated tumor cells [59].

Another pathway involving amino acid metabolism that can be altered by p53 loss is tyrosine metabolism, which has a strong influence on the reprogramming of the solid tumor microenvironment, including nerve fibres. Examination of the sympathetic and parasympathetic branches of the autonomic nervous system showed that fibres positive for tyrosine hydroxylase (TH, adrenergic), but not those positive for vesicular acetylcholine transporter (parasympathetic), are significantly denser in mutant p53 oral cavity squamous cell carcinomas (OCSCCs) than in wild-type p53 tumors [60].

p53 may regulate proline metabolism, which has received considerable attention as a mechanism of NAD/NADP regeneration [61]. p53 transactivates both proline dehydrogenase (PRODH)/proline oxidase (POX) and aldehyde dehydrogenase 4 (ALDH4), which catalyze the first and second reactions of proline degradation, respectively [62]. The regulation of these two enzymes by p53 is thought to be tightly controlled, resulting in distinct effects on the redox state of cells and influencing the overall outcome of p53-mediated responses.

Thus, tight regulation of amino acid metabolism by p53 appears to be important for tumor proliferation through manipulation of the tumor microenvironment, redox state and nutrient limitation.

### p53 and ferroptosis

The abnormal increase in intracellular ′iron′ was discovered in 2012 by treatment with erastin, which causes cell death called ferroptosis [63]. Ferroptosis is an iron-dependent form of non-apoptotic cell death induced by excessive peroxidation of polyunsaturated fatty acids (PUFAs) and reactive oxygen species (ROS) [64]. Ferroptosis is a topic of great interest because of its critical role in tumorigenesis, development and drug resistance. Classical ferroptosis is tightly mediated by glutathione peroxidase 4 (GPX4) [65] and radical trapping antioxidants (RTAs) [66], the former converting lipid hydroperoxides into non-toxic lipid alcohols. GPX4 inhibition has been proposed as a therapeutic strategy to induce ferroptosis and treat cancer [67]. However, the selectivity and potency of GPX4 inhibitors are not consistent across different cancer cells, suggesting that other factors may mediate ferroptosis. Elegant studies have discovered that p53 plays a dual role in ferroptosis, controlling the positive and negative effects of ferroptosis through certain metabolic factors.

p53 increases cell sensitivity to ferroptosis. The first link between p53 and ferroptosis was made when researchers found that the *p533KR* mutant failed to induce cell cycle arrest, senescence and apoptosis, but induced ferroptosis upon ROS-induced stress via SLC7A11, a key component of the cystine/glutamate antiporter [5]. The mutant p53 (mut-p53) with an impaired TAD domain is unable to repress SLC7A11 and promotes ferroptosis [68]. Moreover, p53 modulates an epigenetic mechanism for ferroptosis. p53 negatively regulates the occupancy of histone H2B monoubiquitylation at lysine 120 (H2Bub1) on the SLC7A11 gene regulatory region by promoting the nuclear translocation of the deubiquitinase USP7, resulting in a decrease in SLC7A11 protein levels [69]. In addition, p53 promotes ferroptosis by regulating the expression of SAT1, an enzyme involved in polyamine metabolism that catalyzes the conversion of spermidine and spermine back to putrescine. p53 transcriptionally activates the expression of SAT1, leading to lipid peroxidation and sensitizing cells to undergo ferroptosis, which also leads to suppression of tumor growth in xenograft tumor models [70]. Interestingly, p53 has also been implicated in the suppression of ferroptosis. For example, in human colorectal cancer, p53 inhibits the activity of dipeptidyl peptidase-4 (DPP4) in a transcription-independent manner, thereby inducing the reduction of ferroptosis. DPP4 is mainly located at the plasma membrane where it acts as a serine protease to regulate lipid metabolism [71]. p53 induces the localization of DPP4 to the nucleus, where it acts as a transcriptional cofactor [72]. Stabilization of p53 with the MDM2 inhibitor nutlin-3 delayed the onset of ferroptosis in HT-1080 cells expressing wild-type p53. In contrast, pretreatment with nutlin-3 had weaker effects on erastin2-induced ferroptosis in p53-null H1299 cells and p53-mutant T98G cells [73]. The unstable p53, which is regulated by RRM1 and MDM2, inhibits the activity and expression of GPX4 by repressing the p21 protein [74]. In epithelial ovarian cancer, MEX3A mediates the stability of p53 to suppress ferroptosis and promote tumorigenesis [75], while CUL9 binds to p53 to inhibit erastin-induced ferroptosis [76]. The different cell types and interventions used in these studies and the duality of p53 functions may explain the controversial results of p53 regulation in ferroptosis.

As studies in this field continue to deepen and broaden, new insights into the characteristics of ferroptosis are emerging beyond these old models. In canonical ferroptosis, acyl-CoA synthetase long-chain family member 4 (ACSL4) activity is required for GPX4 inhibition to induce ferroptosis. Beyond canonical ferroptosis, p53 activation modulates the ferroptosis response without affecting GPX4 function. Instead, *ALOX12* knockdown reduces ROS stress-induced p53-mediated ferroptosis and inhibits p53-dependent tumor growth in xenograft models, suggesting that ALOX12 plays a critical role in p53-mediated ferroptosis. Loss of one ALOX12 allele or ALOX12 missense mutations is sufficient to accelerate tumorigenesis in lymphoma models. However, ALOX12 is dispensable for ferroptosis induced by GPX4 inhibitors or erastin. Thus, p53-mediated ferroptosis appears to be dependent on ALOX12, but not on ACSL4 [77]. Furthermore, iPLA2β-mediated detoxification of peroxidised lipids also suppresses p53-mediated ferroptosis in a GPX4-independent manner. In tumor cells, depletion of endogenous iPLA2β renders them susceptible to p53-driven ferroptosis, which in turn promotes p53-dependent tumor suppression in xenograft mouse models. Mechanistically, iPLA2β reduces the levels of peroxidised membrane lipids by inhibiting ALOX12 protein level. Unlike GPX4, loss of iPLA2β does not affect normal tissue development or cell viability in normal tissues, whereas iPLA2β is critical for ROS-induced ferroptosis in tumor cells. Therefore, targeting iPLA2β may be an attractive strategy for the treatment of human cancers without serious toxicity [78].

Nevertheless, given the complex metabolic actions on ferroptosis, it remains necessary to identify if there are other metabolic and non-metabolic processes modulated by p53 that contribute to ferroptosis.

## Metabolism Regulates p53

**Figure 2:**
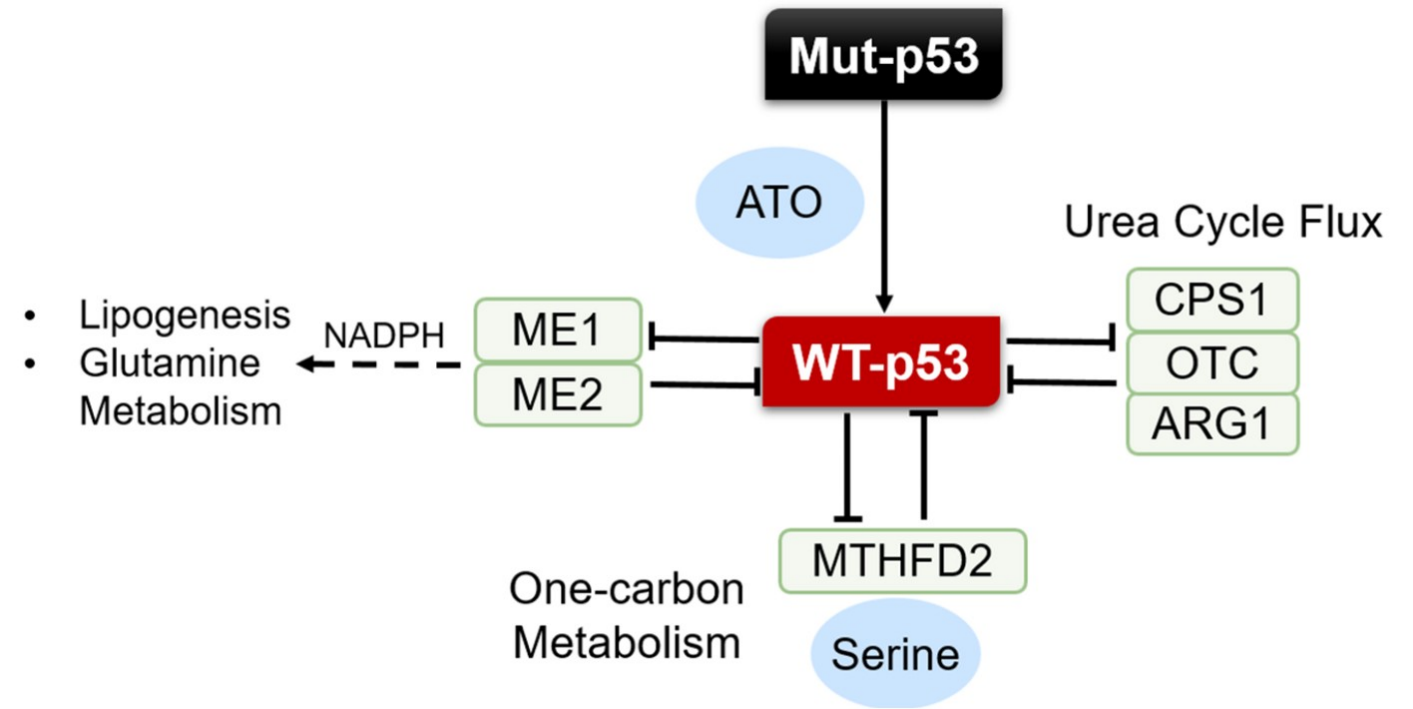
Figure 2 Metabolites play important role in p53 regulation The crosstalk between p53 and ME1/ME2, urea cycle and one-carbon metabolism are necessary for cells to resist stress, such as cancer. Arsenic trioxide (ATO) suppresses tumor by reactivating p53 mutations.

To ensure proper regulation of cellular metabolism, it is essential to have an accurate sense of intracellular nutrient, metabolite and energy status. Elegant studies have shown that metabolism can regulate the stability and activation of p53, meaning that p53 may be an important sensor of nutrient and energy status. We have found that p53 regulates tumor suppression by linking metabolic enzymes and senescence. p53 represses the expressions of the malic enzymes ME1 and ME2, which catalyze the oxidative decarboxylation of malate to pyruvate with the production of NADPH and are important for lipogenesis and glutamine metabolism, to regulate cell metabolism and proliferation. Mutual downregulation of ME1 and ME2 activates p53 in a feed-forward manner through MDM2- and AMP-activated protein kinase-mediated mechanisms, respectively, leading to a strong induction of senescence [26] ( Figure 2).

Furthermore, p53 plays an unexpected role in suppressing ureagenesis and ammonia excretion through transcriptional repression of urea cycle genes such as carbamoyl phosphate synthetase 1 ( *CPS1*), ornithine transcarbamoylase ( *OTC*) and arginase 1 ( *ARG1*). And blocking the urea cycle by downregulating the expression of these metabolic genes activates p53 through an MDM2-dependent mechanism [18]. In addition, the reciprocal regulation of the one-carbon metabolic enzyme MTHFD2 and p53 is critical for tumor cell survival. On the one hand, MTHFD2 acts as a transcriptional target for p53, linking p53 to one-carbon metabolism and dampening cellular reactive oxygen species (ROS). On the other hand, MTHFD2 inhibits p53 activity by reducing AICAR to prevent AMPK activation [59]. In addition, serine is a major source of one-carbon units, and cancer cells are particularly sensitive to deprivation of one-carbon units by serine restriction or inhibition of *de novo* serine synthesis [79]. Indeed, by efficiently channeling depleted serine stores to glutathione synthesis and inducing a transient p21-dependent G1 arrest, p53 promotes cancer cell survival during serine starvation. Cells lacking p53 fail to complete the response to serine deprivation, resulting in oxidative stress, reduced viability and severely impaired proliferation [56].

Metabolites or small molecules sensed by p53 may be excellent p53-targeting drugs. The sensor of arsenic trioxide (ATO) is a star small molecule in the treatment of acute promyelocytic leukemia. It has been reported that ATO is a cysteine-reactive compound that rescues p53 structural mutations. Crystal structures of arsenic-bound p53 mutants reveal a cryptic allosteric site involving three arsenic-coordinating cysteines within the DNA-binding domain, distal to the zinc-binding site. Arsenic binding stabilizes the DNA-binding loop-sheet-helix motif along with the entire b-sandwich fold, conferring thermostability and transcriptional activity to p53 mutants. In cellular and mouse xenograft models, ATO reactivates the 25 most common p53 mutations responsible for tumor suppression, providing an exciting method to overcome cancer containing mutant p53 [80] ( Figure 2).

## Mutant p53 in Cancer Metabolism

The *TP53* gene is the most commonly mutated gene in human cancer. Mutation sites are mainly concentrated in exons 5‒10, and over 80% of p53 mutations occur between amino acids 126‒306, which is the DNA binding domain. p53 mutations include several hot spot mutations that account for 30% of the mutant p53 protein expressed in cancer cells, such as R175, R245, R248, R273 and R282 [ 81, 82] . p53 mutations can be divided into nonsense mutations and missense mutations, the latter not only losing the tumor suppressor function of wild-type p53 (wt-p53), but also gaining new oncogenic functions [83]. According to the different mechanisms by which p53 mutations modulate tumor cell fate, missense mutations in p53 can be divided into two main types: binding defect mutations which can lead to a loss of the p53 protein′s ability to bind to DNA, and conformation mutations which cause structural changes in p53 [84]. In addition to promoting cell proliferation, angiogenesis, migration, invasion, metastasis and chemoresistance, mutant p53 (mut-p53) also alters metabolism to promote tumor development.

Our team recently reported that in addition to regulation between malic enzyme 2 (ME2) and wt-p53, ME2 also cross-talks with mut-p53. mut-p53 protein is stabilized by ME2-generated 2-hydroxyglutarate (2-HG). ME2 depletion decreases cellular 2-HG levels *in vitro* and *in vivo*, which binds directly to mut-p53 and reduces Mdm2-mediated mut-p53 ubiquitination and degradation. 2-HG supplementation is sufficient to maintain mut-p53 protein stability in ME2-depleted cells and restores tumor growth in ME2-depleted cells, but not in cells lacking mut-p53. This suggests that mut-p53 is a sensor of 2-HG, which contributes to mut-p53 stabilization and tumor growth [85].

The genes targeted by wt-p53 and mut-p53 sometimes overlap. In terms of regulation of lipid metabolism, while wt-p53 represses the mevalonate pathway, mut-p53 binds to and activates the transcription factor SREBP1/2, thereby inducing the expressions of a number of genes in the mevalonate pathway and the fatty acid synthesis pathway, thereby promoting tumorigenesis [86]. Interestingly, inhibition of the mevalonate pathway may in turn affect the acquired ability of mutant p53 in tumorigenesis, as blocking the mevalonate pathway can lead to the degradation of mutant p53 protein by inducing CHIP-mediated nuclear export and ubiquitination of mutant p53, resulting in the downregulation of mutant p53 protein levels in cancer cells [87]. AMPK is known to inhibit lipid synthesis and promote fatty acid oxidation by phosphorylating and inhibiting SREBP1/2 and acetyl-CoA carboxylase (ACC) [ 88, 89] . In addition, induction of mevalonate 5-phosphate production along the mevalonate pathway has been shown to increase mut-p53 stability by stimulating its interaction with the Hsp40/DNAJ chaperone, which inhibits mut-p53 ubiquitination [87]. In response to energy stress, mut-p53 binds to AMPKα and inhibits AMPK activation, disrupting the metabolic checkpoint between catabolism and anabolism and increasing invasive cell growth [90]. There is strong evidence that mut-p53 increases the expression of genes involved in fatty acid (FA) synthesis ( *e.g.,* FASN) [91]. Thus, although wt-p53 promotes lipid catabolism, mut-p53 promotes lipid anabolism, which promotes aggressive tumors [92].

Unlike wt-p53, which inhibits glycolysis, p53 mutants can activate glycolysis in cancer cells and promote cancer cell proliferation and growth [93]. Mut-p53 promotes membrane translocation of the glucose transporter GLUT1 by activating the small G protein RhoA and its downstream kinase ROCK. ROCK plays a key role in promoting vesicular trafficking of GLUTs [93]. Inhibition of RhoA/ROCK/GLUT1 can block the function of mut-p53 to promote glycolysis and strongly inhibit the acquired function of mut-p53 to promote tumorigenesis [93]. Notably, by promoting glucose uptake by cancer cells, mut-p53 also inhibits autophagy-dependent protein degradation processes during glucose deprivation [94]. Additionally, mut-p53 has been reported to increase nucleotide synthesis by transcriptionally activating several nucleotide metabolism genes, including RRM2b, DCK and TK1 [95]. With the help of ETS2 recruiting mut-p53 to promoters containing ETS-binding sites, mutant p53 transcriptionally regulate nucleotide metabolism genes, effecting rNTP and dNTP pools [95]. It suggests that upregulation of nucleotide biosynthesis possibly contributes to mut-p53-mediated tumorigenesis.

In addition to the miraculous effects of ATO in reactivating p53 mutations, a number of approaches have been developed in clinical trials to restore the function of mutant p53 [96]. Depending on whether p53 function is inactivated by disrupting direct binding to specific DNA (known as contact mutants) or by preventing the correct folding of the DNA-binding domain of p53 (known as structural mutants) [97], there are different strategies for designing small molecules to restore mutant p53 activity. PRIMA-1 ^MET^, which restores mutant p53 activity (R273H and R175H) by converting mutant p53 to a stable and active form that induces apoptosis in cancer cells, has successfully completed a Phase I clinical trial [ 98‒ 100] . On the other hand, APR-246 induces conformational changes in mutant p53 that restore its DNA-binding activity [99]. Due to the importance of zinc for the proper folding of the central core domain of p53 [101], a number of studies have shown that the addition of zinc can restore the DNA binding activity of the contact mutant p53R273H and the structural mutant p53R175H [102]. PRIMA-1 and APR-246 affect the metabolism of tumor cells. PRIMA-1 can induce oxidative stress and inhibit glycolysis in cancer cells, leading to decreased ATP production and cell death [103]. APR-246 has also been shown to inhibit glycolysis and increase ROS in cancer cells [104].

**Figure 3:**
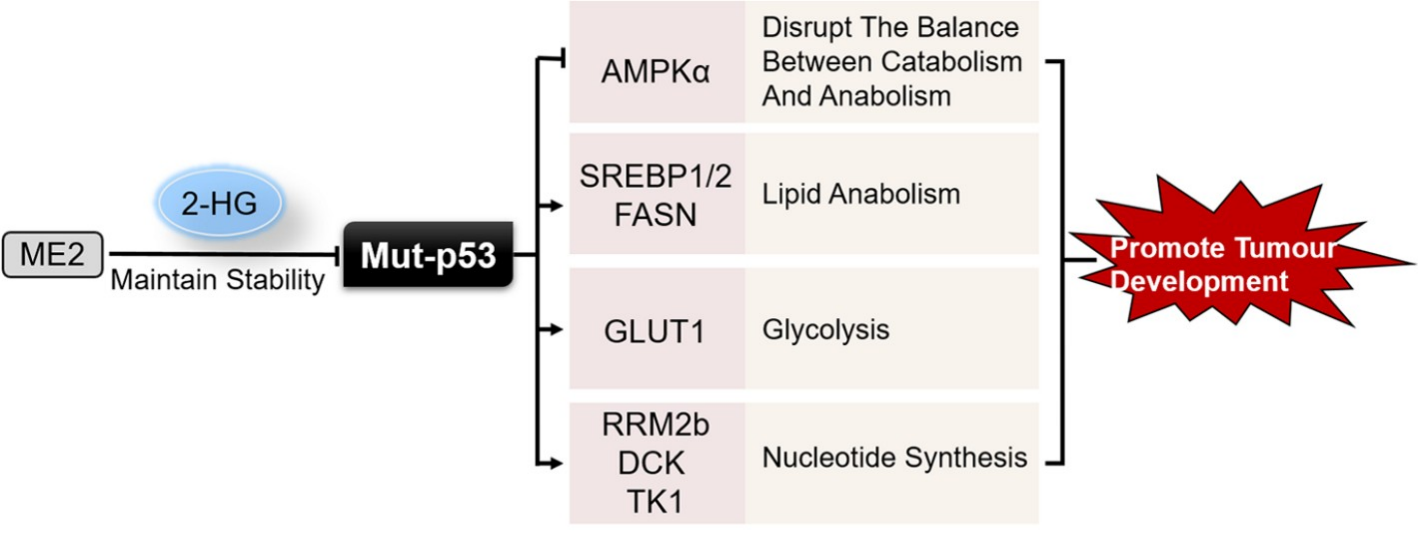
Figure 3 Regulatory role of mut-p53 in metabolism Mut-p53 is a sensor of 2-HG, which can be controlled by ME2. Different from wild-type p53, mut-p53 inhibits AMPKα and boosts lipid anabolism, glycolysis and nucleotide synthesis to promote tumor development.

Taken together, these studies suggest that the new function acquired by mutant p53 to promote metabolic reprogramming of cancer cells may be a key mechanism in tumorigenesis ( Figure 3). In view of this, the development of drugs that act as molecular chaperones to rescue the activity of the mutant p53 would be a promising strategy.

## Conclusions and Future Prospects

Despite more than 40 years of understanding the role of p53 in cancer, there are still several key unanswered questions, including the regulation mechanism in the sleeping guardian p53 and how to wake it up. The last decade has seen a massive expansion in cancer research, including improved experimental models of study, such as metabolite profiling approaches, CRISPR-Cas9 and 3D cell culture models [ 105‒ 108] , and new insights of biology, such as liquid phase separation [109] and oscillations in genes [110]. All of these provide a variety of new ways of thinking about p53, making it possible to gain a deeper understanding of p53. For example, by coupling differential equations and biophysical mechanisms, Mogens H. Jensen found that p53 oscillations increase the efficiency of DNA repair, bridging the gap between the dynamical properties of a transcription factor and the microscopic processes of droplet formation and DNA repair [110]. Methodological improvements will not only improve our understanding of the function of p53 in cancer, but also lead to better therapies for cancer treatment [ 106, 111, 112] . For instance, domain-focused CRISPR screens identified BRD8 as a p53-dependent vulnerability [113], and targeting the bromodomain of BRD8 may be a promising therapeutic strategy for patients with wt-p53 in glioblastoma multiforme (GBM), for which there has been no improvement in treatment for decades. Moreover, the current consensus is that p53 works in concert with a network of implementers to achieve its tumor suppressor function. However, the relative importance of p53 in other aspects of disease, such as diabetes, obesity, nerves and ageing, is much less understood. In addition, the role of p53 in gene editing also needs further investigation and attention. For example, it has been shown that CRISPR-Cas9 genome editing induces a p53-mediated DNA damage response and cell cycle arrest, and that p53 greatly reduces the efficiency of precise genome editing in human pluripotent stem cells (hPSCs) [ 114, 115] .

Finally, although we may think we understand p53 well, the most fundamental question of how p53 suppresses tumors remains unknown, severely limiting the clinical targeting of p53-deficient or mutant tumors. It is also clear that many of p53′s regulatory functions are spatially and temporally specific. Future studies may need to focus more on the behavior and regulatory mechanisms of p53 at specific time points and conditions.
